# Mapping the health science librarianship research field in 2012–2022

**DOI:** 10.29173/jchla29626

**Published:** 2022-12-01

**Authors:** Vinson Li

**Affiliations:** PharmD, MI Student, Dalhousie University; Halifax, NS.

## Abstract

**Introduction:**

Evidence-based practice is an important aspect of health science librarianship. However, good evidence-based practice can only occur if the body of evidence is also of adequate quality. By using bibliometric techniques to map the health science librarianship research field, one can better understand the properties of the evidence base in health science librarianship.

**Methods:**

The Library Literature & Information Science Full Text database was used to generate a bibliography of publications pertaining to health librarianship limited to the time span of 2012–2022. Using Excel and Microsoft Power BI, a descriptive analysis was conducted. VosViewer was used to create a subject term co-occurrence map.

**Results:**

The average number of publications per year is 207.3 and it was trending downwards for 2012–2022. The most frequently assigned subject term was “survey”. The average number of authors per paper is 2.5 and was trending upwards. The subject term co-occurrence map identified 5 clusters of keywords, which were interpreted as major themes found in the body of literature.

**Discussion:**

The 5 keyword clusters were interpreted as major themes found in the body of literature. The identified themes were professional development, measuring the value output of librarian services, measuring the return on investment of library resources, improving the quality of LIS research, and outreach to other library and healthcare institutions. This depicts the health science librarianship research landscape as one of collaboration, concerned with finding ways of demonstrating value, and connecting with other types of libraries and the public.

## Introduction

A good medical practice is supported by evidence from research. While it would be ideal for healthcare professionals to stay current with emerging research, the volume of health science research is simply too much for any one person to keep up. Thus, there is a need to curate and summarize all this information into manageable quantities. This can take many forms, such as knowledge syntheses, libguides, literature reviews, and more. Healthcare librarians play major roles in these efforts. The work of healthcare librarians directly impacts the practice and research of healthcare professionals which in turn impacts the health of everyone [1].

The publishing of textbooks and guidelines lag behind the publishing of primary literature, and with a field as fast-paced as healthcare, a few years of research can make all the difference. This sort of environment is why it was only natural that the concept of evidence-based practice (EBP) should first arise from the field of medicine [2]. EBP became popular due to its robust persuasiveness in providing immediate benefit to patient care [2].

Due to their proximity with healthcare professionals, health science librarians pioneered the adoption of EBP in the field of librarianship [3]. EBP extends beyond healthcare; EBP encompasses the idea that decision-making should be informed by evidence. We should always ask the question: is there anything in the literature that supports your decision? [4]. Of course, research in medicine and librarianship are very different, and the types of questions asked are also different [3]. Nonetheless, EBP is still applicable to librarianship because it makes for more persuasive pitches to stakeholders and aids in predicting future trends and demands [4].

However, good EBP can only occur with a good body of research on which to base practice [1]. Librarians recognized this and many endeavors were undertaken to help support better EBP. One way to improve EBP is to promote library and information science (LIS) research; the Journal of the Medical Library Association (JMLA) focused on providing research relevant to practice, and the journal Evidence Based Library and Information Practice was created for the same reasons [1].

Others have made attempts to describe and quantify the health science librarianship body of research in order to illuminate its overall state. Myers preformed an analysis on the Medical Library Association (MLA) annual meeting abstracts 2001–2019 and found that discussions have shifted toward a more user-focused service design [6]. Funk did a textual analysis of the articles from JMLA from 1961–2010 to empirically chart out the history of the library profession noting the gradual deemphasis on physical collections over time among other trends [7]. Ul-Haq conducted a bibliometric analysis of the LIS journal Science & Technology Libraries articles from 1980–2020 and found that while the volume of publications had decreased during that time span, the citation counts had gone up inferring a greater impact on the librarianship field [8]. Ul-Haq et al. also analyzed the publications in The Journal of Hospital Librarianship from 2001–2020 and used keyword frequency analysis to understand the thematic trends within the journal [9]. They have all conducted these studies to have a better understanding of the LIS field, its research, and its trends from the past and present. Understanding what the body of research currently offers will inform what kinds of EBP are possible and spotlight research gaps and opportunities.

No other research has been found that have attempted to map out the health science librarianship research field at the database level. Therefore, this paper aims to provide some insight into research trends in the health science librarianship field. The hope is that the knowledge generated from this paper will provide health science librarians a chance to have an informed reflection on the research that has been done in the field, and to give insight into neglected areas for future research.

### Objectives of the study

The purpose of this study is to examine the trends in the body of health librarianship research from 2012–2022. A ten-year time span was chosen to provide a more contemporary snapshot of the research field. The trends will be illustrated by answering the following questions:

1. How many publications related to health science librarianship were published during 2012–2022?
2. What topics or subjects are most popularly written about? Do any greater themes emerge from the subject terms tagged in the articles?
3. Which journals dominate in the health science librarianship field?
4. What does authorship collaboration look like in health science librarianship research?

While experts in health science librarianship can provide opinions into trends in the field and its research, an analysis such as this provides a more objective insight that supplements expert opinions and overall informs librarians about the state of the research body and how it can be improved.

## Methods and data

### Extracting the set of publications from the database

The body of health science librarianship literature was extracted from the Library Literature & Information Science Full Text database. This EBSCO database provides access to 213 indexed and abstracted journals on topics relevant to library and information science as well as other topics such as government aid, censorship, automation, public relations, and more [10]. The date coverage of the database stretches back to 1980 [10].

Scopus was not used because nearly half the publications in the query did not have assigned keywords making parts of the analysis unviable. A broader more multidisciplinary database such as Academic Search Premier was tried, but there were too many irrelevant results, so it was not used. Therefore, Library Literature & Information Science Full Text was used in the end for its specificity even if some comprehensiveness was sacrificed.

The following search query was used to compile the set of publications:

((DE “Medical libraries” OR DE “Dental libraries” OR DE “Hospital libraries” OR DE “Nursing libraries” OR DE “Pharmaceutical libraries” OR DE “Public health libraries” OR DE “Veterinary libraries” OR DE “Medical librarians” OR DE “Medical library reference services”) OR (DE “Medical librarianship”)) OR (TI ( “medic* _o-occu*” or “health _o-occu*” or “health science* _o-occu*” ) OR AB ( “medic* _o-occu*” or “health _o-occu*” or “health science* _o-occu*” ) OR SU ( “medic* _o-occu*” or “health _o-occu*” or “health science* _o-occu*” ))

The publication dates were limited to January 1, 2012–March 23, 2022. An RIS file of all 4093 results were exported onto Zotero. From Zotero, the collection was re-exported, this time as a CSV file. Each row represented a single publication. The most relevant headings were: publication ID number, publication year, authors (separated by semicolon), journal title, publication title, abstract, and subject terms (separated by semicolon).

### Analysis using Power BI and Excel

Microsoft Power BI Desktop [11] was used to transform the data as well as perform some of the analyses. The CSV data’s subject term column was delimited using semicolons so that each subject term for each article had its own row (i.e. if an article had 3 subject terms, there will now be 3 identical rows of data except the subject term column will have the three subject terms).

Excel [12] was also used to transform the data and perform other analyses. While both programs have the ability to perform all the analyses and data transformations required, some were more easily done on one program.

### Analysis using VosViewer

VosViewer [13] is a software tool that can be used to create bibliographic networks. In this study, it was used to generate a subject term network. The network consists of nodes and links between said nodes. In this case, the nodes represent a subject term (e.g. medical libraries or survey). If two subject terms are tagged in one publication, then a link is formed between the two. For every subsequent publication where those two subject terms are used, the link grows stronger and the distance between the nodes is reduced. The size of the node also represents how often a subject term was used in the body of literature. Ul-Haq performed this type of analysis to map out the keyword co-occurrence network for articles in the journal Science & Technology Libraries [8].

When the whole network is generated, some patterns may emerge. Some subject terms may be associated with each other more so than with others, possibly because they have more to do with each other. For example, “YA literature”, “children’s programming”, and “Roblox” might be more linked to each other than to “medical library association”, which would make sense since those first three terms are related to public libraries. VosViewer’s clustering algorithm is able to identify these patterns and highlights these clusters in various colors.

For this analysis, the RIS file was used. A cooccurrence of keywords (i.e. subject terms) was selected, and a full count method was chosen. In order to reduce crowding and improve readability in the visualization, a subject term had to appear in at least 44 publications for it to be used in the network. 80 subject terms met this threshold. The number chosen was somewhat arbitrary; essentially different parameters were chosen until a visualization was obtained that was readable and informative.

## Results

The following sub-sections will discuss the results of the analyses done using Power BI, Excel, and VosViewer.

### Publications statistical analysis

**Fig. 1:**
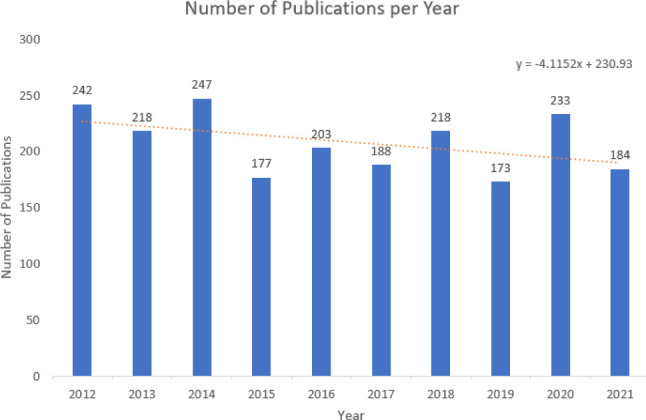
Publications per year

A bar chart with publication year and count of publications was used to determine how many publications were published per year (Figure 1). A trendline was added to emphasize changes in publication volumes over the decade. Because the bibliographic data was extracted in March 2022, the true number of publications that will be published in 2022 has yet to be realized which is why 2022 was omitted. Excluding 2022, the average number of publications per year is 207.3. The slope of the trendline is negative meaning that there appears to be a slight downward trend in the number of publications per year.

### Subject terms statistical analysis

A count of all subject terms displayed on a bar chart and sorted by greatest to least was done to show the most frequently used subject terms. Due to the nature of the search query used to find the set of publications, practically all publications would have subject terms such as “medical libraries” and “medical librarianship”. Those sorts of subject terms were filtered out of this analysis as it would dwarf all the other subject terms and provide no insight. **Error! Reference source not found**. shows a list of all the subject terms filtered for this analysis.

**Table 1:** List of filtered subject terms

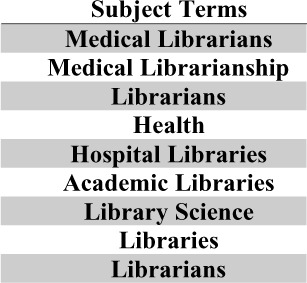

**Fig. 2:**
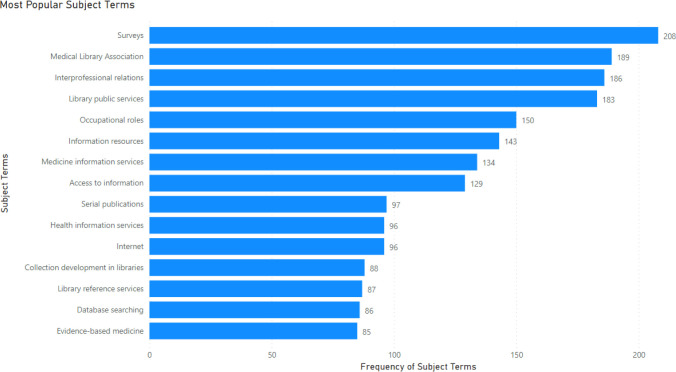
Most frequently used subject terms

Figure 2 shows the top 15 most used subject terms assigned to the publications in the database from 2012–2022. The most popular subject term was “survey”

### Author statistical analysis

The number of authors was counted for each publication, and a line graph that shows the average number of authors per publication for each year was created (**Error! Reference source not found**.). 2022 was omitted since the year was not over at the time of publication. By counting the number of authors per paper, it has been determined that the average number of authors per paper is roughly 2 in the beginning of the decade, but has slowly risen to about 2.5 at the end of the decade.

**Fig. 3:**
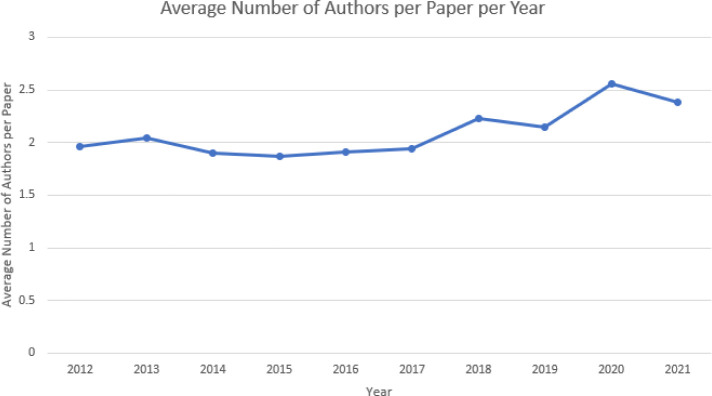
Mean number of authors per paper from 2012–2021

### Journal statistical analysis

The total number of publications in the dataset was 2093. **Error! Reference source not found**. is a donut chart showing the distribution of publications within the most popular journals in the database. The most popular journal is JMLA, but it does not contain the clear majority of publications. **Error! Reference source not found**. shows the number of publications per journal with the top 12 journals distinguished from the others.

**Fig. 4:**
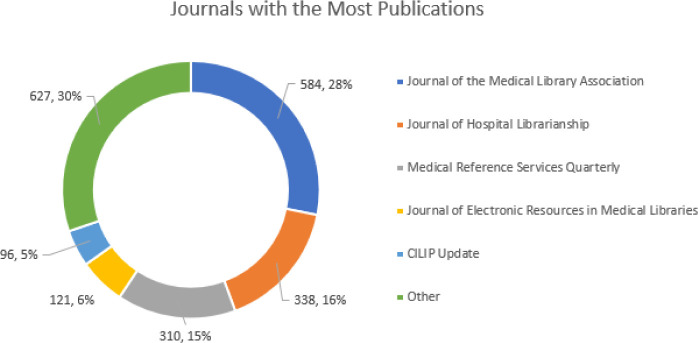
Number of publications per journal

**Table 2:** Highly published journals

Journal Title	Number of Publications
Journal of the Medical Library Association	584
Journal of Hospital Librarianship	338
Med Ref Serv Q	310
Journal of Electronic Resources in Medical Libraries	121
CILIP Update	96
Journal of Consumer Health on the Internet	62
Against the Grain	33
Library Philosophy & Practice	32
Evidence Based Library & Information Practice	31
American Libraries	24
College & Research Libraries News	22
Library Journal	19
Other	404
Total	2076

### Subject term network map

While the default setting of the network map has the nodes depicted as circles and the links as lines between the circles, the round shape of the health science librarianship research map made that view difficult to read. Thus, a density cloud view was chosen for legibility.

**Fig. 5:**
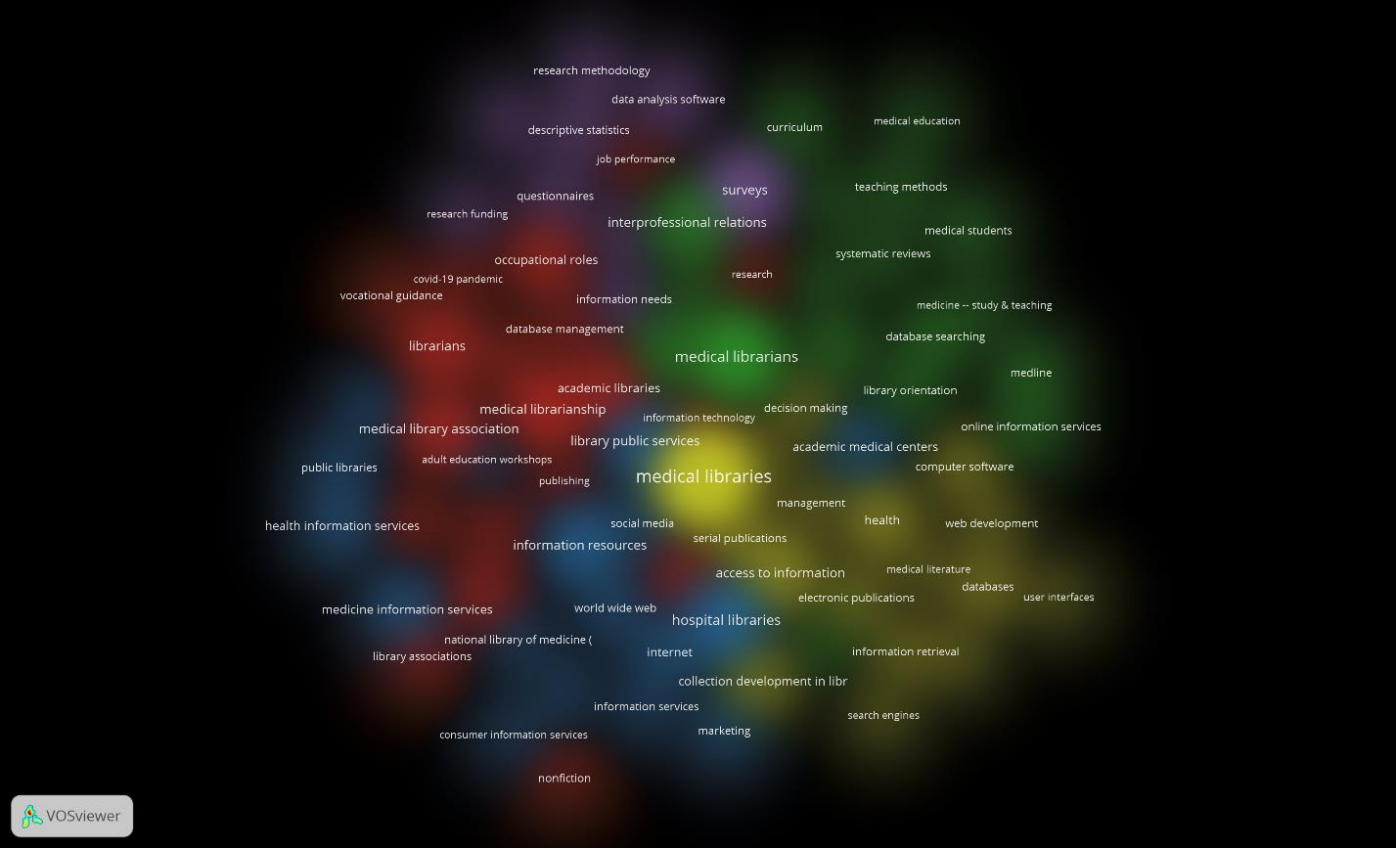
Subject term co-occurrence network map

Figure 5 shows the subject term co-occurrence network map. Five clusters were identified. By examining the subject terms that fall under each cluster, a theme for each cluster can be identified.

The purple cluster has terms that talk about research methods with terms like “survey”, “descriptive statistics”, and “research methodology”.

The red cluster has terms related to the health science library profession and career advice such as “vocational guidance”, “occupational roles”, and “library associations”.

The yellow cluster speaks to libraries as a location and the resources it has to offer with terms such as “computer software”, “collection development in libraries”, and “access to information”.

The green cluster is about the services that health librarians provide with terms such as “systematic reviews”, “library orientation”, and “medical education”.

The blue cluster has terms related to health science librarianship outside of academic library contexts such as “hospital libraries”, “public libraries”, and “adult education workshops”.

Table 3 shows all the subject terms on the network grouped by cluster. Note that not all subject terms fit cleanly into the prescribed themes mentioned above. Also note that many clusters have subject terms that are exclaves within other clusters such as “job performance” being part of the red cluster but is located within the purple cluster. This is possibly because “job performance” can pertain to career advice but could also describe a metric for a research method.

**Table 3:** Subject terms grouped by cluster

*Cluster*	*Red*	*green*	*blue*	*yellow*	*purple*
***Search Terms***	academic libraries	college teachers	academic medical centers	access to information	communication
	conferences & conventions	curriculum	adult education workshops	collection development in libraries	data analysis software
	covid-19 pandemic	database searching	consumer health services	computer software	descriptive statistics
	database management	evidence-based medicine	health information services	databases	information needs
	information technology	human services program	health literacy	decision making	interviewing
	job performance	information literacy	hospital libraries	electronic publications	questionnaires
	librarians	information storage & retrieval	information resources	health	research funding
	libraries	interprofessional relations	information services	information resources management	research methodology
	library associations	library orientation	internet	information retrieval	surveys
	library reference services	medical education	library public services	management	
	library science	medical librarianship	marketing	medical libraries	
	medical librarianship	medical research	medicine information services	medical literature	
	medical library association	medical students	national library of medicine (u.s.)	search engines	
	nonfiction	medicine --study & teaching	patient education	serial publications	
	occupational roles	medline	public libraries	user interfaces	
	professional employee training	online information services	social media	web development	
	publishing	systematic reviews	world wide web	wireless communications	
	research	teaching methods			
	vocational guidance				
***Total:***	19	18	17	17	9

## Discussion

This study looked at the trends in health science librarianship research from 2012–2022 using the Library Literature & Information Science Full Text database. An examination into the volume of publications generated by the field shows that there has been a slight decline in the past decade. A possible explanation may be an increase in publisher quality specifications reducing the number of publications compared to prior years [8]. The average number of authors per paper was 2 and has risen to about 2.5 by the end of the decade which may mean an increase in research collaboration within the field. This is supported by Ul-Haq’s analysis of the journal Science & Technology Libraries which saw an increase in collaborative authorship during the same decade [8]. An analysis of authorship collaboration in the Annals of Library and Information Studies, it was found that most of the papers has 2 authors and that collaboration was increasing over the 2010s [14]. The most popular journal in the field is the JMLA which makes sense as it is one of the main hubs of disseminating health science librarian research [4].

The most prominent subject term that was not directly related to libraries was “survey”. This aligns with what Marshall’s team found when they examined all articles published in the Bulletin of the Medical Library Association and JMLA from 1961 to 2010 for research-related terms; one of which was “survey” [3]. Also, a lot of librarian research uses surveys as part of their methods when conducting research [5].

On the five themes identified in the network map, be mindful that individual publications are not necessarily siloed into a single cluster. A single publication’s keywords may be linked across several clusters meaning the publication likely touches on multiple themes. Some publications may fall predominantly into one cluster while others were evenly distributed throughout. The five clusters possibly speak to major concerns within the health librarian field which will be interpreted below.

The green cluster that focused on a theme of services that the health science library offers may have emerged partly due to the fact that libraries these days have to demonstrate value and impact leading to publications of that nature trending [15]. The mainstream uptake of online search engines led to hospitals to reconsider the value of hospital libraries leading to the closure of many hospital libraries [16]. Thus, hospital libraries everywhere had to demonstrate to their stakeholders that they do indeed provide value that a simple search engine cannot, necessitating the creation of continuing education courses related to value demonstration [16]. Ultimately, self-preservation is an interest that affects every single health science librarian and it is sensible that such a theme would arise in the analysis.

Related to the idea of librarians and the services they provide and how that demonstrates added value is the more traditional metric for demonstrating value using citation analysis, and library resource usage reports that show a return on investments [15]. While nowadays, emphasis is on services from the librarians themselves is gaining prominence, showing how much use a library’s resources get is important to show administrators that the institution’s assets are useful to their faculty and students. This is even more important in the current climate of budget cuts. So important is the analysis of resource use that models and standards have been developed and the use of such standards have also been a topic of research [17, 18]. These concerns map well onto the subject terms in the yellow cluster which was identified as being related to a library’s physical assets.

Beyond value demonstration, health science librarians also conduct research that speaks to career development. Professional development is important for health science librarians, but exactly what that should look like is a major topic of research and discussion which is a possible explanation for the red cluster that speaks about the profession and career advice [19]. Articles of this nature could discuss guidance for librarians new to the health sciences [20], forecasting new roles and trends emerging in the profession [21], and even the implementation of equity, diversity, and inclusion into health science librarianship [22].

The purple cluster on research methods may be due to librarians discussing the methods and methodologies of conducting research in the health science librarianship field [23]. It could also include aspects about the quality of research health librarians produce and the role that different levels of evidence play in evidence-based librarianship [24]. Included in this cluster were even some criticisms about the overuse of surveys in LIS research methods [25].

The blue cluster which generally concerned affairs outside of the academic library was more difficult to summarize and is more diverse than the other clusters. This is reflected in the shape of the blue cluster on the network map being less centralized, having exclaves, and branches reaching out into the other clusters. To better understand what concerns the blue cluster handles, the articles that have blue cluster subject terms were examined for commonalities. There are aspects of public library – health science library partnerships [26], hospital librarians and their interactions with clinicians [27], the library’s role in providing consumer health information [28], and more. The blue cluster is unique in that it’s very interconnected with other fields, while the other four clusters are very inward facing, only looking at health librarianship.

### Limitations and future steps

This paper has by no means comprehensively analysed the health science librarianship research field, as there are a number of limitations that need to be acknowledged. This analysis only focused on one specialized database and focused on subject term analysis. Further analyses in other databases, such as Scopus, could be used to conduct a citation analysis on the field as a whole.

While the body of published literature in the librarianship field is important to EBP, grey literature also plays a pivotal role [29]. For example, library conference presentations and their presentation slides provide a wealth of information. However, grey literature does not make a clean dataset, so the body of literature was restricted to only published works with properly indexed bibliographies.

Finally, the cluster map data could be used to visualize how all the publications distribute across the five themes using a scatterplot with 5 axes arranged like a pentagon. Additionally, the dataset also provided title and abstract data; thus, natural language processing might provide further insights that the subject terms cannot.

## Conclusions

Health science librarianship has a longer relationship with EBP than any other LIS sub-field due to its proximity to its conception [3]. It recognizes that a good body of research is required to have good EBP [1]. From the analysis conducted in this study, the body of health librarian research is depicted as an increasingly collaborative landscape that is concerned with professional development, measuring the value output of librarian services and library resources, improving the quality of LIS research, and outreach to other library and healthcare institutions. By having this insight, this author hopes that other health science librarians may think about what is missing and what can be done.

## Data Set Availability

The bibliographic data used for the analysis is available as a CSV and RIS file on the Open Science Framework (OSF) using the link below. The TXT files required to recreate the subject term network on VosViewer is also available on OSF using the link below.

https://osf.io/8xhd6/
